# Inactivation of Thioglucosidase from *Sinapis alba* (White Mustard) Seed by Metal Salts

**DOI:** 10.3390/molecules25194363

**Published:** 2020-09-23

**Authors:** Monika Marcinkowska, Henryk H. Jeleń

**Affiliations:** Faculty of Food Science and Nutrition, Poznań University of Life Sciences, Wojska Polskiego 31, 60-624 Poznań, Poland; monika.marcinkowska@up.poznan.pl

**Keywords:** myrosinase, thioglucosidase, enzyme inactivation, glucosinolates hydrolysis, kohlrabi, isothiocyanates, nitriles, SPME

## Abstract

The glucosinolates which are specialized plant metabolites of *Brassica* vegetables are prone to hydrolysis catalyzed by an endogenous enzyme myrosinase (thioglycoside hydrolase, thioglucosidase) that exists in Brassica plant tissue causing volatile isothiocyanates release. Currently existing literature data on the inactivation of myrosinase is insufficient in particular for use in the analysis of volatile and odor compounds in vegetables rich in glucosinolates. In this study, the impact of different metal salts in effective inactivation of enzyme activity was investigated by solid-phase microextraction (SPME) and GC/MS system in aqueous samples and kohlrabi matrix. A saturated solution of calcium chloride which is commonly used to stop enzyme activity in plant tissue inactivates the myrosinase–glucosinolate system. However, even without the participation of myrosinase, it changes the reaction pathway towards nitrile formation. The model experiment shows that optimum efficiency in inhibition of the enzyme system shows iron(III) ions, silver ions, and anhydride sodium sulfate resulting in no volatile products derived from glucosinolates. However, in the kohlrabi matrix, the strongest enzyme inhibition effect was observed for silver salt resulting in no volatile products, also both anhydrous Na_2_SO_4_ and saturated CaCl_2_ solution seem to be useful inhibitors in flavor studies.

## 1. Introduction

Enzymes (mainly myrosinase and lipoxygenase) play a crucial role in the creation of Brassica vegetable flavor. Myrosinase can convert glucosinolates into volatile products that not only contribute to the aroma of vegetables, but also have documented health-promoting properties (anticarcinogenic activity, chemoprotective, prevention of cardiovascular and neurodegenerative disorders) [1]. The lipoxygenase enzyme family provide catalytic oxidation of polyunsaturated fatty acids with a *cis*,*cis*-1,4-pentadiene moiety resulting in a number of important metabolites and odor active compounds [2,3]. Meat-like flavors associated mainly with Maillard reaction can be created by a double enzyme (Flavourzyme) in the two-step hydrolysis of Brassica proteins [4]. Knowledge of enzymes allows for controlling the enzymatic processes in plant tissue and thus maintain the quality by using a wide range of anti-browning agents against enzymatic browning of fruit and vegetable products [5].

On the other hand, enzymes pose an analytical challenge, especially in plant-derived food. The study which is based on analysis of vegetable tissues is enormously complex due to variable content of compounds which are specific for plant species, varieties, environmental conditions, part of a plant, cultivation, the region of origin, the status of growth, the season of cultivation, postharvest, and food processing [6], and varying activity of endogenous enzymes such as myrosinase [7]. Myrosinase is endogenous thioglucoside glucohydrolase that is present in glucosinolate-rich *Brassicaceae* vegetables such as broccoli, brussels sprout, cabbage, cauliflower, kohlrabi, mustard, and radish; however it was also found in bacteria, fungi and even in the human gut microflora [8]. Glucosinolates in presence of myrosinase are prone to hydrolysis after tissue disruption (blending, cutting) into β-d-glucose and an intermediate product (aglycon). This latter compound is spontaneously converted into isothiocyanates, nitriles, thiocyanates, epithionitriles, indoles, or 1,3-oxazolidine-2-thiones. The type of glucosinolate product obtained during hydrolysis strictly depends on the reaction conditions and the original glucosinolate side-chain structure (aliphatic, aromatic, and indolic) [9,10].

Myrosinase is an enzyme that converts glucosinolates into glucosinolate degradation compounds, most of which are volatile and odor active. An active enzymatic reaction in the vegetable tissue is an analytical problem as it leads to inaccurate results and large variations between samples. Screening of volatiles is not the only branch of analytical chemistry where blocking of thioglucosidase enzyme is important. Quantitative analysis of metabolites (glucosinolates) in plant tissues requires the enzyme to be blocked, which prevents their further degradation during the analysis and leads to deviations in the obtained results. The inactivation of the enzyme at an exact time is also important to increase the reproducibility of results obtained between laboratories. 

Currently, there are only few studies about the inactivation methods of myrosinase. The only well-known approach of deactivating this enzyme activity is pH changing [11], thermal treatment [12], pressure enzyme inactivation, or fusion of these latter two methods [13]. However, both methods have disadvantages; the first approach cannot be used simultaneously with the analysis of thermolabile compounds and pressure inactivation needs specific equipment that is unique and not available for all laboratories. 

The aim of this study was to investigate the impact of different metal salts in inactivation of myrosinase activity. The utilization of metal salts is simple to use, widely available, and relatively cheap. This knowledge will be useful for future research in flavor studies especially for identification and quantification of volatile compounds extracted from plant tissue and to improve analytical extraction protocols for glucosinolate-containing plants by increasing reproducibility when preparing samples for analysis.

## 2. Results

### 2.1. Preliminary Experiments with Commercial Thioglucosidase

**Figure 1:**
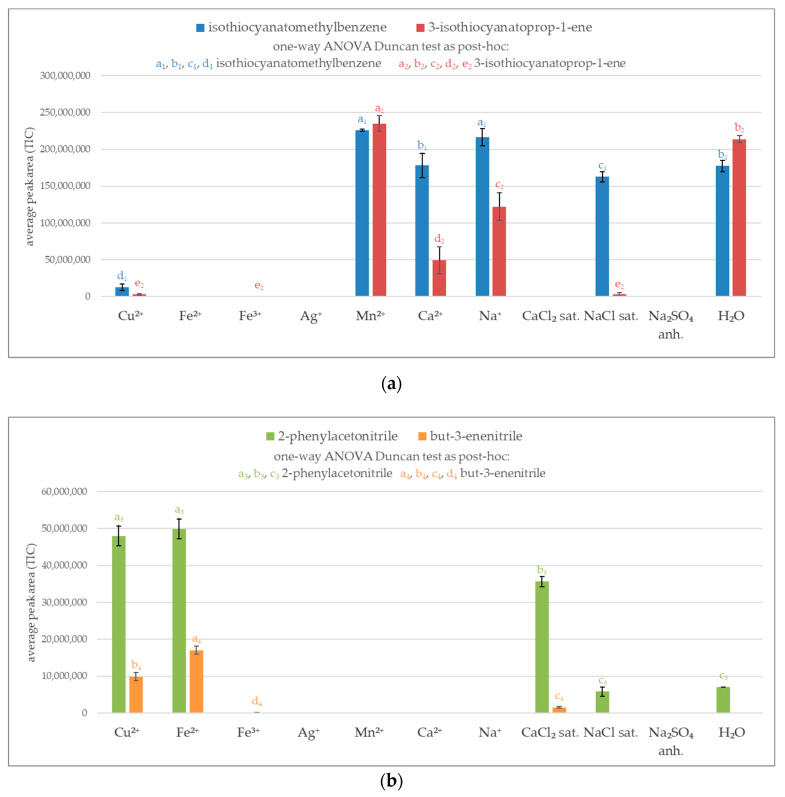
Volatile products of glucosinolates breakdown in miscellaneous enzyme inactivation media; sat.—saturated solution; anh.—anhydrous; ^a, b, c, d, e^—the result of statistical analysis (one-way ANOVA Duncan test as post-hoc), values followed by the same letter in a Duncan grouping are not significantly different, the subscript number and letter color are corresponding to the chart legend (α = 0.05); (n = 3); (**a**) Isothiocyanates (isothiocyanatomethylbenzene derived from glucotropaeolin and 3-isothiocyanatoprop-1-ene is a product from sinigrin); (**b**) Nitriles (2-phenylacetonitrile from glucotropaeolin and but-3-enenitrile from sinigrin).

A preliminary experiment relied on analyzing volatile glucosinolate breakdown products in aqueous system. To select the most effective agent for the inactivation of thioglucosidase, the volatile products were determined by GC/MS system. The peak area represents the total ion current (TIC). As shown in Figure 1a not all of the investigated metal salts were effective at blocking myrosinase, resulting in a similar amount of obtained isothiocyanates as in the control test (H_2_O). Manganese ions proved to be completely ineffective at blocking the enzymatic reaction for both glucosinolates, glucotropeaolin, and synigrin. The adequate molar concentration of calcium and sodium ions and saturated solution of sodium chloride exhibited inhibition effect for synigrin, nevertheless they were ineffective in the case of blocking glucotropeaolin hydrolysis.

The maximum efficiency in inhibition of enzyme system was noticed for iron ions (Fe^2+^ and Fe^3+^), silver ions, saturated solution of calcium chloride, and anhydrous sodium sulfate. The use of these salts did not produce volatile isothiocyanates for both glucotropeaolin and synigrin. However, some of the potential enzyme-blocking agents produced volatile nitriles, for example iron(II) and saturated calcium chloride. Except for these salts, copper(II) ions show the ability to produce a considerable amount of nitriles (Figure 1b).

**Figure 2:**
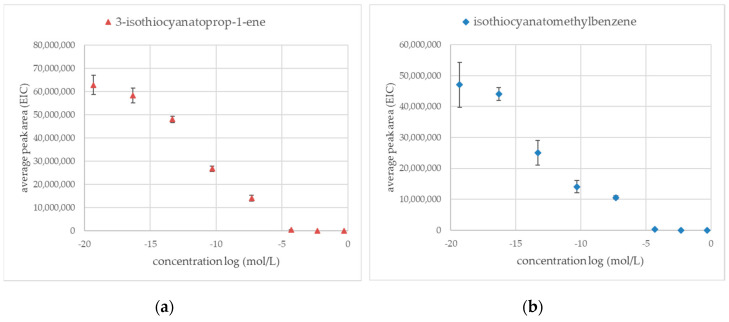
Area of the peaks (EIC) to the logarithm of the concentration of silver ions in the aqueous solution (mol/L); Statistical analysis: One-way ANOVA Duncan test as post-hoc (α = 0.05); (n = 3) (**a**) 3-isothiocyanatoprop-1-ene derived from sinigrin (*m*/*z* = 99.2); values obtained for concentration log in the range of 0 to −5 are not significantly different (**b**) Isothiocyanatomethylbenzene derived from glucotropaeolin (*m*/*z* = 91.2); values obtained for concentration log in the range of 0 to −5, −7, to −11, and −15 to −20 are not significantly different.

Previous preliminary experiments show that silver ions were one of the most effective methods to inactivate myrosinase enzymatic activity resulting in no volatile products derived from glucosinolate. Figure 2a shows silver ions dependent inhibition of sinigrin hydrolysis curve where concentration is presented as a logarithm function. The same experiment was performed on glucotropeaolin glucosinolate (Figure 2b). The average peak areas were measured in extracted-ion chromatogram mode (EIC) for products of enzymatic hydrolysis: 3-isothiocyanatoprop-1-ene (*m*/*z* = 99.2) and for isothiocyanato methylbenzene (*m*/*z* = 91.2).

The silver ions were an effective inhibition factor for the inactivation of thioglucosidase for both glucosinolate sinigrin and glucotropeaolin. The lowest concentration of silver salt, which is effective in blocking thioglucosidase enzyme activity was 5 × 10^−5^ mol/L. Results obtained for the lowest investigated concentration of silver salt (5 × 10^−20^ mol/L) equals average peak areas measured for the control sample (water, enzyme, sinigrin, or glucotropeaolin).

Results obtained from the first experiment indicated that the favored volatile products for some enzyme-blocking agents (metal salts) are nitriles. An investigation of the interaction between glucosinolate and inactivation agent to investigate non enzymatic reaction pathway was performed. To this stage, metal salts were selected with the best ability in blocking enzymatic hydrolysis of glucosinolates. In that part of the study, volatile compounds after the addition of glucosinolate to an aqueous solution of metal salts without enzyme (thioglucosidase from *Sinapis alba*) addition were measured. The control sample was water and glucosinolate (sinigrin potassium salt or glucotropaeolin potassium salt) in which no volatiles were produced.

**Figure 3:**
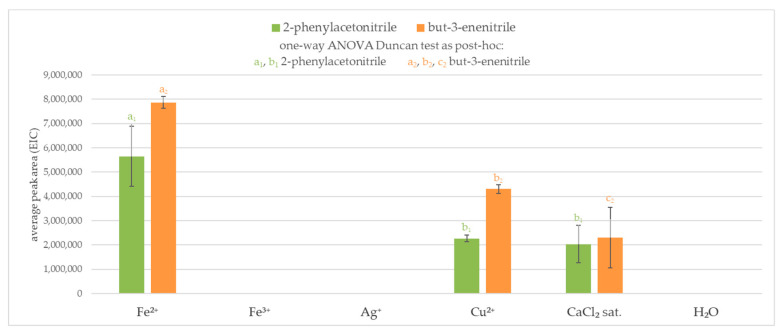
Volatile products from the interaction between glucosinolate (sinigrin or glucotropaeolin) and an enzymatic reaction blocking agent (without enzyme presence); 2-phenylacetonitrile (*m*/*z* = 117.2); but-3-enenitrile (*m*/*z* = 41.3); ^a, b, c^—the result of statistical analysis (one-way ANOVA Duncan test as post-hoc), values followed by the same letter in a Duncan grouping are not significantly different, the subscript number and letter color are corresponding to the chart legend (α = 0.05); (n = 3).

It was observed that aqueous silver and iron(III) solutions did not result in any volatile products derived by chemical degradation of glucosinolates. On the other hand, iron(II) and copper(II) ions or saturated solution of calcium chloride produced nitriles (2-phenylacetonitrile and but-3-enenitrile). When both sinigrin and glucotropaeolin were used, the highest amount of nitrile was obtained in the iron(II) solution. In a blocking agent environment, more volatile products were formed from sinigrin than glucotropaeolin from the same amount of glucosinolate, nevertheless, results obtained in saturated sodium chloride solution were similar. The results are presented in Figure 3.

### 2.2. Study of the Effect of Metal Salts on the Native Enzyme in Kohlrabi Tissue

**Figure 4:**
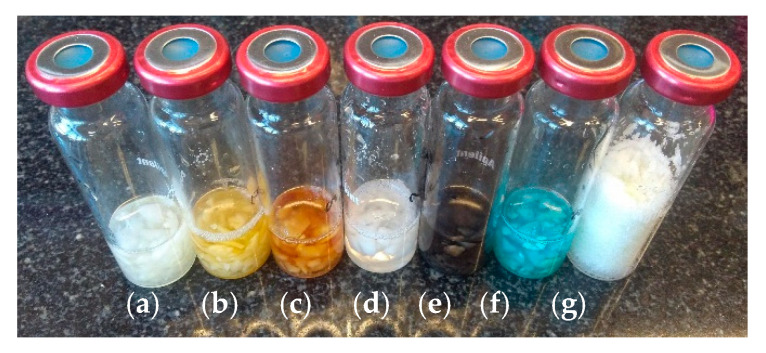
Kohlrabi matrix samples with addition of sinigrin potassium salt after GC/MS analysis (**a**) control (H_2_O); (**b**) iron(II) sulfate (FeSO_4_); (**c**) iron(III) sulfate (Fe_2_(SO_4_)_3_); (**d**) saturated solution of calcium chloride (CaCl_2_); (**e**) silver nitrate (AgNO_3_); (**f**) copper sulfate (CuSO_4_); (**g**) anhydrous sodium sulfate salt (Na_2_SO_4_).

There is no doubt that plant tissue is a different environment than water, that is why the behavior of glucosinolates (absent in kohlrabi and used previously in the model experiment) was investigated in a model matrix in which this class of compounds occur naturally. The same batches of kohlrabi were used for established volume (3 mL) of aqueous metal salts solutions or sufficient amount of anhydride sodium sulfate salt. The control sample was water and glucosinolate (sinigrin potassium salt or glucotropaeolin potassium salt) without any metal salt addition. Compared to the model experiment, a larger volume of salt solution was used—allowing the complete immersion of plant tissue in the aqueous solution and an adequately increased amount of glucosinolate. It was observed that kohlrabi samples after extraction and analysis differed depending on the metal salt used for the analysis. Results of GC/MS analyses are shown in Figure 4.

The main volatiles derived from glucosinolates in various environmental conditions in the kohlrabi matrix are presented in Table 1. But-3-enenitrile was formed only in samples with addition of synigrin with the presence of iron(II) and copper(II) ions. The most abundant peak for 3-isothiocyanatoprop-1-ene was recorded in control sample (H_2_O), low amounts of this isothiocyanate were also noted for a saturated solution of calcium chloride and anhydrous sodium sulfate. Iron(III) and silver ions caused the complete blockage of the enzyme that hydrolyzes the added synigrin.

The presence of 2-phenylacetonitrile was not recorded in the sequence with the addition of synigrin, while it was detected in the samples with the addition of glucotropaeolin. The highest amount of 2-phenylacetonitrile was found for copper(II) ions, half less when iron(II) ions were used, and negligible amounts were found in the calcium chloride and iron(III) solutions. Isothiocyanatomethylbenzene was also found only in the samples to which glucotropaeolin was added. However, the presence of this isothiocyanate was not confirmed in the samples with the addition of synigrin. The only peak for isothiocyanatomethylbenzene was in the control sample (H_2_O).

The profile of 4-methylsulfanylbutanenitrile and 5-methylsulfanylpentanenitrile average peak area depending on various environmental conditions for samples with the addition of synigrin and glucotropeaoline was similar. The higher content of 4-methylsulfanylbutanenitrile than the control was observed in both samples with the addition of sinigrin and glucotropaeolin in aqueous solutions of copper(II) sulfate, and iron(II) salts. In aqueous solution of silver salt 4-methylsulfanylbutanenitrile was not found. Concerning the control sample, an increased amount of this compound is observed in the presence of copper ions, while in the medium of silver and iron(III) ions, the presence of this nitrile was not observed.

For the 1-isothiocyanato-3-methylsulfanylpropane and 1-isothiocyanato-4-methylsulfanylbutane, the average peak area values follow a comparable trend. Isothiocyanates were observed only in the control sample (H_2_O), in a saturated solution of sodium chloride and anhydrous sodium sulfate. Average peak areas for the 1-isothiocyanato-4-methylsulfanylbutane are analogous, and for 1-isothiocyanato-3-methylsulfanylpropane peak area obtained for samples with saturated sodium chloride solution is slightly reduced compared to the control sample.

Miscellaneous volatiles not related to the breakdown of glucosinolates were shown in Table 2. Addition of silver ions resulted in no volatiles produced. Sulfides ((methyldisulfanyl)methane and (methyltrisulfanyl)methane) were obtained mainly in the control sample and samples with anhydrous sodium sulfate and iron(II) sulfate. The presence of benzaldehyde was found in almost all tests except those with an aqueous solution of silver nitrate and anhydrous sodium sulfate. Compared to the control sample, an increased concentration of volatile compounds (hexanal, 2-pentylfuran, heptan-2-one, (E)-non-2-enal) was observed with the use of iron(III) ions. The highest mean peak value for hexanal was observed for iron(III) ions, copper ions, and iron(II) ions, respectively. Pent-1-en-3-ol was detected in tests with the addition of iron(II) ions, saturated sodium chloride solution and anhydrous sodium sulfate.

## 3. Discussion

The composition of Brassica vegetable tissue usually promotes the formation of isothiocyanates [19]. Glucosinolates are prone to hydrolysis by myrosinase and as a result of degradation various volatile compounds can be obtained, which are strictly dependent on the conditions (e.g., pH value, availability of iron(II) (Fe^2+^) and the presence of specific proteins that interact with myrosinase) [20,21]. Several heavy metals were reported to exhibit inactivation of enzymatic reaction on different types of enzymes that are used especially in biosensors studies [22]. Saturated sodium chloride and calcium chloride solutions are known to inactivate enzyme system and they are commonly used in flavor study [23,24]. An alternative method to stop the enzyme reaction is to remove water by using anhydrous sodium sulfate [25]. However, myrosinase has so far been poorly studied, particularly concerning the volatile analysis and flavor study.

Results of the preliminary experiments showed that the addition of metal salts may have different consequences such as decreasing or stopping of enzyme activity, change of breakdown pathways, or salting-out effect. Silver and iron(III) ions are enzyme reaction inhibitors as heavy metals. Silver ions completely block the action of myrosinase, even at a concentration of 5 × 10^−5^ mol/L, regardless of the glucosinolate used. Their action can be explained by the conformational change of myrosinase protein. Anhydrous sodium sulfate is also effective because it removes water without which the enzyme is inactive. A saturated solution of sodium chloride is a valuable inhibitor concerning aliphatic glucosinolates however, in the case of aromatic glucosinolates it is ineffective. A slight inhibitory effect with a similar tendency was observed in equimolar solutions of sodium and calcium ions. This effect can be explained by changing the enzyme’s active center, which blocks the enzyme’s activity only in relation to a specific substrate—the group of aliphatic glucosinolates.

The increased average peak area of isothiocyanates in samples with additional manganese salt is related to the salting-out effect. It is well known that iron(II) ions as well as the acidic environment promote the formation of nitriles, which is explained by the formation of a complex with glucosinolate. Copper also forms considerable amounts of nitriles because, like iron(II), it is a transition metal that willingly forms supramolecular structures. What is more, both the aqueous solution of iron(II) and copper(II) are acidic (pH < 7), because their salts are derived from strong acids and transition metals. Nevertheless, the previously tested aqueous solution of manganese chloride does not change the reaction pathway to nitriles, which suggests that when using transition metal salt ions, the formation of the complex is a more important factor than the pH value of the reaction medium. Interestingly, a saturated sodium chloride solution indeed blocked the possibility of obtaining isothiocyanates, but nitriles were recorded instead. The use of saturated sodium chloride solution to inactivate the enzyme may falsely increase the proportion of nitriles in the analysis of volatile compounds directly from vegetables in the sample. Metal salts which do not form any volatile compounds such as isothiocyanates and nitriles from the glucosinolate can be considered as a suitable blocking agent.

An experiment with glucosinolate and selected metal salts was executed to show that nitriles can be formed without the enzyme presence. The highest amount of nitriles was obtained when iron(II) ions were used, which is consistent with current scientific reports [26,27]. The presence of nitriles was also noted in samples with copper ions and a saturated sodium chloride solution. The absence of the enzyme in the samples tested suggests that the degradation of glucosinolates can be non-enzymatic. Thus, the degradation of glucosinolates can take place catalytically through the formation of a complex structure [26]; however, the full confirmation of this hypothesis still requires further research.

Measurements of myrosinase activity are usually done by utilizing isolated enzyme and water environment however to establish the best blocking agent for myrosinase it is needed to study this phenomenon in cellular conditions [28]. But-3-enenitrile and 3-isothiocyanatoprop-1-ene are formed from synigrin. 2-phenylacetonitrile and isothiocyanatomethylbenzene are obtained from glucotropaeolin. These two glucosinolates were artificially added to the vegetable. Additionally, kohlrabi contains native glucosinolates (glucoerucin, glucoiberverin, glucoraphanin [29]) which form the corresponding volatile products (4-methylsulfanylbutanenitrile, 1-isothiocyanato-3-methylsulfanylpropane, 1-isothiocyanato-4-methylsulfanylbutane). 5-methylsulfanyl pentanenitrile derived from glucoberteroin.

In kohlrabi bulb tissue several volatiles were observed that are related to the action of lipoxygenase (e.g., hexanal, (E)-hex-2-enal, pent-1-en-3-ol, (E)-hex-2-enal, 2-pentylfuran, heptan-2-one, (Z)-hex-3-en-1-ol, (E)-non-2-enal) [30], therefore, we can additionally evaluate the effectiveness of the metal salts used to block this enzyme. According to the preliminary experiment, the strongest inhibition effect of thioglucosidase inactivation in the kohlrabi matrix has a silver and iron(III). However, the iron(III) salt causes an important increase in some volatile compounds derived from lipid oxidation, primarily hexanal, 2-pentylfuran and heptan-2-one, which may distort the results of the analyzes (profile of volatiles). Enzyme inactivation can be caused by changing the conformation of the enzyme protein and destroying the key and lock enzyme system. Likewise, in the kohlrabi matrix environment of copper and iron(II) salts, analogous products are formed as in the model system. The change of the pathway of glucosinolates degradation by these salts is most likely caused by the catalytic decomposition of an intermediate complex compound.

## 4. Materials and Methods

### 4.1. Materials and Reagents

The reference compounds (3-isothiocyanatoprop-1-ene (CAS 57-06-7), but-3-enenitrile (CAS 109-75-1), isothiocyanatomethylbenzene (CAS 622-78-6), 2-phenylacetonitrile (CAS 140-29-4)) of the highest available GC purity (>98%) were obtained from Sigma-Aldrich (Poznań, Poland). Thioglucosidase from *Sinapis alba*, sinigrin potassium salt, anhydrous sodium sulfate, iron(II) sulfate heptahydrate, SPME fiber divinylbenzene/ carboxen/polydimethylsiloxane (DVB/CAR/PDMS) were purchased from Sigma-Aldrich (Poznań, Poland), glucotropaeolin potassium salt was purchased from Extrasynthese (Genay Cedex, France), copper(II) sulfate pentahydrate was purchased from Chempur (Piekary Śląskie, Poland) and silver nitrate was purchased from POCh S.A. (Gliwice, Poland). Fresh kohlrabi was purchased at a local grocery store (Poznań, Poland).

### 4.2. Preliminary Experiments with Commercial Thioglucosidase from Sinapis alba: Screening of Potential Metal Inhibitors

One milliliter of liquid was added (0.5 mol/L of Cu^2+^_(aq)_, Fe^2+^_(aq)_, Fe^3+^_(aq)_, Ag^+^_(aq)_, Mn^2+^_(aq)_, Na^2+^_(aq)_, and Ca^2+^_(aq)_, saturated solutions of NaCl, CaCl_2_ or water) was placed in a 20 mL vial. 50 µL of aqueous thioglucosidase from *Sinapis alba* (2.9 g/L) was added to the vial. Afterward 50 µL of an aqueous solution of glucosinolate (2 mmol/L of sinigrin potassium salt or glucotropaeolin potassium salt) was added and the vial was immediately closed with a cap. Fifty microliters of aqueous thioglucosidase from *Sinapis alba* (2.9 g/L) was added to the vial. A sufficient amount of anhydride Na_2_SO_4_ was placed in a 20 mL vial. Afterward 50 µL of an aqueous solution of glucosinolate (2 mmol/L of sinigrin potassium salt or glucotropaeolin potassium salt) was added and the vial was immediately closed with a cap.

The stock solution of silver nitrate (0.5 mol/L AgNO_3_) was diluted step by step to obtain the following concentrations: 5 × 10^−2^, 5 × 10^−3^, 5 × 10^−4^, 5 × 10^−5^, 5 × 10^−6^, 5 × 10^−8^, 5 × 10^−11^, 5 × 10^−14^, 5 × 10^−17^, 5 × 10^−20^ mol/L. One milliliter of silver solution was placed in a 20 mL vial and 50 µL of an aqueous thioglucosidase from *Sinapis alba* (2.9 g/L) was added to the vial. Subsequently, 50 µL of aqueous solution of glucosinolate (2 mmol/L of sinigrin potassium salt or glucotropaeolin potassium salt) was added and the vial was immediately closed with cap.

1 mL of liquid was added (0.5 mol/L of Cu^2+^_(aq)_, Fe^2+^_(aq)_, Fe^3+^_(aq)_, Ag^+^_(aq)_, and saturated solution of CaCl_2_) was placed in a 20 mL vial. 50 µL of an aqueous solution of glucosinolate (2 mmol/L of sinigrin potassium salt or glucotropaeolin potassium salt) was added and the vial was immediately closed with a cap. These tests were negative control, while the positive control sample was prepared with 1 mL of water and adequate glucosinolate addition.

### 4.3. Endogeneous Thioglucosidase Activity of Kohlrabi Extract and Application of Metal Inhibitors

Kohlrabi bulb with no water added was homogenized using a table-top stainless steel blender. 2 g of kohlrabi was placed in a 20 mL vial. The enzymatic reaction blocking agent (Cu^2+^_(aq)_, Fe^2+^_(aq)_, Fe^3+^_(aq)_, Ag^+^_(aq)_, anhydrous Na_2_SO_4_, and saturated solution of CaCl_2_) was added whenever in the exact time (1 min and 30 sec) after blending and glucosinolates addition (200 µL of 2 mmol/L of sinigrin potassium salt or glucotropaeolin potassium salt). The vial was immediately closed with a cap. The control sample was prepared with 2 g of kohlrabi, 3 mL of water, and adequate glucosinolate addition.

### 4.4. Solid-Phase Microextraction and GC-MS Analysis

Extraction of volatiles by SPME and their subsequent analysis were performed on gas chromatograph coupled with triple quadrupole mass spectrometer (7890A/7000B, Agilent Technologies, Santa Clara, CA, USA) working in a single quadrupole mode. The GC/MS system was equipped with an automatic MultiPurpose MPS-2 sampler (Gerstel, Germany). The headspace solid-phase microextraction (SPME) was performed at 40 °C and the sample was incubated for 5 min prior to extraction, and then the fiber was exposed in the vial for 30 min. After extraction SPME fiber was removed from the vial and subsequently was placed in a desorption port (230 °C) of GC-MS system. Samples were injected in a splitless mode.

A Supelcowax-10 column (60 m × 0.25 mm × 0.25 µm, Supelco, Bellefonte, PA) was used and a constant flow of He as a carrier gas was kept at 1 mL/min. The oven temperature was 40 °C for 1 min, ramped at 8 °C/min to 200 °C and 20 °C/min to 280 °C, and held at 280 °C for 5 min. The transfer line temperature was 260 °C. The mass spectrometer was operating in an electron ionization (EI) mode at 70 eV, in a scan range of *m*/*z* 33–333. The temperature of MS ion source was 230 °C. Instrument control, data acquisition, and peak area count calculation were performed by Agilent Technologies MassHunter Workstation (B 07.00, Agilent Technologies, Santa Clara, CA, USA) software with a library of the National Institute of Standards and Technology (version 2.0) (NIST, Gaithersburgh, MD, USA). Then the spectra of volatile compounds derived from synigrin and glucotropaeolin were compared with those obtained after the reference compounds analysis. All samples were run in triplicate (n = 3).

## 5. Conclusions

A saturated solution of calcium chloride and anhydrous sodium sulfate salts were effective in inhibiting synigrin and glucotropaeolin hydrolysis in the vegetable matrix. Compared to the preliminary experiment in aqueous system, no major discrepancies were observed for a saturated sodium chloride solution and the formation of an increased amount of nitriles compared to the control. The discrepancies in results obtained from the preliminary experiment and during the use of the vegetable matrix were mainly due to the differences between the chemical composition of these two environments.

Summing up, the most effective inhibitors are silver ions concerning both myrosinase and lipoxygenase deactivation. Milder blocking conditions, resulting in a similar profile of volatile compounds in the control provides the use of anhydrous sodium sulfate.

## Figures and Tables

**Table 1:** Volatile compounds derived from glucosinolates in variable conditions within the kohlrabi tissue.

Compound Name	RRI_exp_	RRI_lit_	H_2_O	Fe^2+^	Ag^+^	Fe^3+^	CaCl_2_ sat. ^1^	Cu^2+^	Na_2_SO_4_ anh. ^2^
sinigrin potassium salt									
but-3-enenitrile	1169	1166 [14]	84767 ^@,b^	1081935 *^,a^	16794 ^#,b^	-	52008 ^#,b^	1231764 ^$,a^	23012 ^$,b^
3-isothiocyanatoprop-1-ene	1356	1383 [15]	21509589 ^#,a^	-	-	68156 ^$,b^	390208 ^$,b^	59443 ^@,b^	257598 ^$,b^
4-methylsulfanylbutanenitrile	1785	1733 [16]	482607 ^#,b^	658725 *^,b^	-	390468 ^#,b^	540572 ^$,b^	979739 ^#,a^	459707 ^#,b^
5-methylsulfanylpentanenitrile	1948	1891 [15]	664729 *^,c^	-	-	814106 ^$,b^	1155252 ^$,b^	2520488 ^#,a^	893718 ^#,bc^
1-isothiocyanato-3-methylsulfanylpropane	1976	1983 [17]	6175537 ^#,a^	-	-	24958 ^@,b^	5770687 *^,a^	-	6790716 ^#,a^
1-isothiocyanato-4-methylsulfanylbutane	2167	2135 [16]	4351726 *^,a^	-	-	-	4149057 ^#,a^	-	4135976 ^#,a^
glucotropaeolin potassium salt									
4-methylsulfanylbutanenitrile	1785	1733 [16]	414374 ^#^^,d^	588352 *^,bc^	-	376014 ^#,d^	561198 ^$,b^	936512 *^,a^	470188 ^#,cd^
2-phenylacetonitrile	1931	1912 [18]	-	1699090 *^,b^	-	114952 ^#,c^	124882 *^,c^	3746581 ^#,a^	-
5-methylsulfanylpentanenitrile	1948	1891 [15]	687165 ^#,b^	-	-	814106 ^$,b^	888626 *^,b^	2335283 *^,a^	679047 ^$,b^
1-isothiocyanato-3-methylsulfanylpropane	1976	1983 [17]	6697967 ^#,ab^	-	-	-	5169479 *^,b^	26058 *^,c^	7497730 ^$,a^
1-isothiocyanato-4-methylsulfanylbutane	2167	2135 [16]	4277184 ^#,a^	-	-	-	3682303 *^,a^	-	3509385 ^$,a^

^1^: saturated solution of calcium chloride; ^2^: anhydrous sodium sulfate; RRI_exp_: relative retention index calculated by n-alkane series using the experimental value (Supelcowax-10 column); RRI_lit_: relative retention index calculated by n-alkane series using the literature value; relative standard deviation: *: RSD ≤ 10%, ^#^: 10% < RSD ≤ 25%, ^$^: 25% < RSD ≤ 50%, ^@^: RSD > 50%; values express the mean peak area; ^a, ab, b, bc, c, cd, d^: the result of statistical analysis (one-way ANOVA Duncan test as post-hoc), values followed by the same letter in a Duncan grouping are not significantly different (α = 0.05); (n = 3).

**Table 2:** Miscellaneous volatile compounds from kohlrabi tissue.

Compound Name	RRI_exp_	RRI_lit_	H_2_O	Fe^2+^	Ag^+^	Fe^3+^	CaCl_2_ sat. ^1^	Cu^2+^	Na_2_SO_4_ anh. ^2^
sinigrin potassium salt									
(methyldisulfanyl)methane	1068	1064 [15]	317715 ^#,b^	69214 ^#,c^	-	5106 ^$,c^	-	2482 ^$,c^	419816 ^$,a^
hexanal	1075	1082 [18]	47284 ^$,b^	169473 ^#,b^	-	3789656 ^#,a^	42992 *^,b^	233815 ^@,b^	61231 ^#,b^
pent-1-en-3-ol	1141	1158 [18]	5905 ^$,c^	119966 *^,c^	-	-	70899 ^$,b^	-	187903 ^#,a^
(E)-hex-2-enal	1218	1216 [18]	-	-	-	-	13660 ^@,b^	-	36592 ^#,a^
2-pentylfuran	1222	1232 [18]	-	26154 *^,b^	-	138788 ^#,a^	-	2988 ^#,c^	2493 ^$,c^
heptan-2-one	1325	1182 [18]	-	-	-	242172 *^,a^	-	-	-
(Z)-hex-3-en-1-ol	1364	1380 [18]	-	10077 ^@,b^	-	-	102469 *^,c^	-	58909 ^#,a^
(methyltrisulfanyl)methane	1391	1376 [18]	2974175 ^#,a^	-	-	-	-	-	83767 ^$,b^
benzaldehyde	1530	1518 [18]	46994 ^$,a^	1311 ^#,c^	-	25629 *^,b^	16560 ^#,b^	2234^#,c^	-
(E)-non-2-enal	1539	1535 [18]	20479 *^,b^	-	-	147120 ^#,a^	-	-	-
glucotropaeolin potassium salt									
(methyldisulfanyl)methane	1068	1064 [15]	615854 *^,a^	93952 ^#,c^	-	-	3438 *^,c^	3848 ^$,c^	342112 *^,b^
hexanal	1075	1082 [18]	71835 ^#,b^	140440 ^$,b^	-	3789656 ^#,a^	27612 ^#,b^	202812 *^,b^	57077 ^#,b^
pent-1-en-3-ol	1141	1158 [18]	9007 *^,b^	100647 *^,b^	-	-	190000 ^$,a^	2830 ^#,b,^	159107 ^$,a^
(E)-hex-2-enal	1218	1216 [18]	-	-	-	-	69644 ^#,a^	-	39481 ^$,a^
2-pentylfuran	1222	1232 [18]	-	10856 *^,b^	-	138788 ^#,a^	-	-	-
heptan-2-one	1325	1182 [18]	-	-	-	242172 *^,a^	-	-	-
(Z)-hex-3-en-1-ol	1364	1380 [18]	-	3675 ^$,b^	-	-	142612 ^#,a^	-	45657 ^$,b^
(methyltrisulfanyl)methane	1391	1376 [18]	3243687 ^#,a^	-	-	-	-	-	88756 ^$,b^
benzaldehyde	1530	1518 [18]	43684 ^#,a^	3781 *^,c^	-	25629 *^,b^	23531 ^#,b^	18571 *^,bc^	-
(E)-non-2-enal	1539	1535 [18]	29224 *^,b^	-	-	147120 ^#,a^	-	-	-

^1^: saturated solution of calcium chloride; ^2^: anhydrous sodium sulfate; RRI_exp_: relative retention index calculated by n-alkane series using the experimental value (Supelcowax-10 column); RRI_lit_: relative retention index calculated by n-alkane series using the literature value; relative standard deviation: *: RSD ≤ 10%, ^#^: 10% < RSD ≤ 25%, ^$^: 25% < RSD ≤ 50%, ^@^: RSD > 50%; values express the mean peak area; ^a, b, bc, c^: the result of statistical analysis (one-way ANOVA Duncan test as post-hoc), values followed by the same letter in a Duncan grouping are not significantly different (α = 0.05); (n = 3).

## References

[1] Bell L., Oloyede O.O., Lignou S., Wagstaff C., Methven L. (2018). Taste and Flavor Perceptions of Glucosinolates, Isothiocyanates, and Related Compounds. Mol. Nutr. Food Res..

[2] Terp N., Göbel C., Brandt A., Feussner I. (2006). Lipoxygenases during Brassica napus seed germination. Phytochemistry.

[3] Baysal T., Demirdöven A. (2007). Lipoxygenase in fruits and vegetables: A review. Enzym. Microb. Technol..

[4] Guo X., Tian S., Small D.M. (2010). Generation of meat-like flavourings from enzymatic hydrolysates of proteins from Brassica sp.. Food Chem..

[5] Moon K.M., Kwon E.-B., Lee B., Kim C.Y. (2020). Recent Trends in Controlling the Enzymatic Browning of Fruit and Vegetable Products. Molecules.

[6] Sikorska-Zimny K., Beneduce L. (2020). The glucosinolates and their bioactive derivatives in *Brassica*: A review on classification, biosynthesis and content in plant tissues, fate during and after processing, effect on the human organism and interaction with the gut microbiota. Crit. Rev. Food Sci. Nutr..

[7] Yábar E., Pedreschi R., Chirinos R., Campos D. (2011). Glucosinolate content and myrosinase activity evolution in three maca (*Lepidium meyenii* Walp.) ecotypes during preharvest, harvest and postharvest drying. Food Chem..

[8] del Carmen Martinez-Ballesta M., Carvajal M. (2015). Myrosinase in Brassicaceae: The most important issue for glucosinolate turnover and food quality. Phytochem. Rev..

[9] Kołodziejski D., Piekarska A., Hanschen F.S., Pilipczuk T., Tietz F., Kusznierewicz B., Bartoszek A. (2019). Relationship between conversion rate of glucosinolates to isothiocyanates/indoles and genotoxicity of individual parts of Brassica vegetables. Eur. Food Res. Technol..

[10] Abellán Á., Domínguez-Perles R., Moreno D., García-Viguera C. (2019). Sorting out the Value of Cruciferous Sprouts as Sources of Bioactive Compounds for Nutrition and Health. Nutrients.

[11] Yen G.-C., Wei Q.-K. (1993). Myrosinase activity and total glucosinolate content of cruciferous vegetables, and some properties of cabbage myrosinase in Taiwan. J. Sci. Food Agric..

[12] Dal Prá V., Jardim N.S., Dolwitsch C.B., Mazutti M.A., Viana C., Bohrer D., Nascimento P.C., Carvalho L.D., Silva M.D., Carvalho C.D. (2013). A review of influence of environment and process parameters on glucosinolate-myrosinase system from Brassica. J. Appl. Pharm. Sci..

[13] Okunade O.A., Ghawi S.K., Methven L., Niranjan K. (2015). Thermal and pressure stability of myrosinase enzymes from black mustard (Brassica nigra L. W.D.J. Koch. var. nigra), brown mustard (*Brassica juncea* L. Czern. var. juncea) and yellow mustard (*Sinapsis alba* L. subsp. maire) seeds. Food Chem..

[14] Zhou Q., Jia X., Yao Y.-Z., Wang B., Wei C.-Q., Zhang M., Huang F. (2019). Characterization of the Aroma-Active Compounds in Commercial Fragrant Rapeseed Oils via Monolithic Material Sorptive Extraction. J. Agric. Food Chem..

[15] Chen W., Karangwa E., Yu J., Xia S., Feng B., Zhang X., Jia C. (2017). Characterizing Red Radish Pigment Off-Odor and Aroma-Active Compounds by Sensory Evaluation, Gas Chromatography-Mass Spectrometry/Olfactometry and Partial Least Square Regression. Food Bioprocess Technol..

[16] Miyazawa M., Maehara T., Kurose K. (2002). Composition of the essential oil from the leaves of Eruca sativa. Flavour Fragr. J..

[17] Kroener E.-M., Buettner A. (2018). Sensory-Analytical Comparison of the Aroma of Different Horseradish Varieties (Armoracia rusticana). Front. Chem..

[18] Babushok V.I., Linstrom P.J., Zenkevich I.G. (2011). Retention Indices for Frequently Reported Compounds of Plant Essential Oils. J. Phys. Chem. Ref. Data.

[19] Hanschen F.S., Klopsch R., Oliviero T., Schreiner M., Verkerk R., Dekker M. (2017). Optimizing isothiocyanate formation during enzymatic glucosinolate breakdown by adjusting pH value, temperature and dilution in Brassica vegetables and Arabidopsis thaliana. Sci. Rep..

[20] Román J., Castillo A., Mahn A. (2018). Molecular Docking of Potential Inhibitors of Broccoli Myrosinase. Molecules.

[21] Marcinkowska M., Jeleń H.H. (2020). Determination of the odor threshold concentrations and partition coefficients of isothiocyanates from Brassica vegetables in aqueous solution. LWT.

[22] Bachan Upadhyay L.S., Verma N. (2013). Enzyme Inhibition Based Biosensors: A Review. Anal. Lett..

[23] Buttery R.G., Teranishi R., Ling L.C. (1987). Fresh tomato aroma volatiles: A quantitative study. J. Agric. Food Chem..

[24] Steinhaus M. (2020). Chapter 9 Gas Chromatography–Olfactometry: Principles, Practical Aspects and Applications in Food Analysis. Advanced Gas Chromatography in Food Analysis.

[25] Steinhaus M. (2015). Characterization of the Major Odor-Active Compounds in the Leaves of the Curry Tree *Bergera koenigii* L. by Aroma Extract Dilution Analysis. J. Agric. Food Chem..

[26] Hanschen F.S., Lamy E., Schreiner M., Rohn S. (2014). Reactivity and Stability of Glucosinolates and Their Breakdown Products in Foods. Angew. Chem. Int. Ed..

[27] Hanschen F.S., Kühn C., Nickel M., Rohn S., Dekker M. (2018). Leaching and degradation kinetics of glucosinolates during boiling of Brassica oleracea vegetables and the formation of their breakdown products. Food Chem..

[28] Verkerk R., Dekker M. (2004). Glucosinolates and Myrosinase Activity in Red Cabbage (*Brassica oleracea* L. Var. *Capitata* f. *rubra* DC.) after Various Microwave Treatments. J. Agric. Food Chem..

[29] Carlson D.G., Daxenbichler M.E., Vanetten C.H., Kwolek W.F., Williams P.H. (1987). Glucosinolates in crucifer vegetables: Broccoli, brussels sprouts, cauliflower, collards, kale, mustard greens, and kohlrabi. J. Am. Soc. Hortic. Sci..

[30] Molteberg E., Magnus E., BJ J., Nilssoni A. (1996). Sensory and Chemical Studies of Lipid Oxidation in Raw and Heat-Treated Oat Flours. Cereal Chem..

